# Explainable artificial intelligence reveals divergent learning in pharmacophore-based hierarchical pooling graph neural networks

**DOI:** 10.1038/s41598-026-59947-0

**Published:** 2026-06-29

**Authors:** Maria Julia Teja Urrutia, Andrea Mastropietro, Jürgen Bajorath

**Affiliations:** 1 Department of Life Science Informatics and Data Science, B-IT, LIMES Program Unit Chemical Biology and Medicinal Chemistry, University of Bonn, Friedrich-Hirzebruch-Allee 6, 53115 Bonn, Germany; 2 Lamarr Institute for Machine Learning and Artificial Intelligence, University of Bonn, Friedrich-Hirzebruch-Allee 6, 53115 Bonn, Germany; 3 Data Science Center, Nara Institute of Science and Technology, 8916-5 Takayama-cho, Ikoma, Nara 630-0192 Japan

**Keywords:** Hierarchical pooling, Graph neural networks, Molecular property prediction, Pharmacophore features, Explainable artificial intelligence, Network learning behavior, Computational biology and bioinformatics, Mathematics and computing

## Abstract

Hierarchical pooling is a promising mechanism to enhance graph neural networks (GNNs) by enabling multi-scale representation learning. Rationalization of hierarchical GNN predictions remains an underexplored area. In this work, we investigate the impact of hierarchical pooling on GNNs for molecular property prediction. We designed architectural variants integrating pharmacophore features with pooling GNNs at different levels. GNN models with pharmacophore-based graph reduction or hierarchical pooling achieved comparable compound classification performance. Explainable artificial intelligence (XAI) methods were applied to compare feature importance and substructure attribution for the different model architectures. Qualitative and quantitative analyses of the resulting explanations demonstrated that the GNN variants had different internal learning characteristics. GNN models based on reduced graphs matched the prediction accuracy of models based on complete graph representations following different variant-dependent learning strategies.

## Introduction

Molecular property prediction based on chemical structure is an integral part of drug design. While standard machine learning approaches using property descriptors are widely used, deep representation learning provides an alternative, aiming to further increase the ability to capture complex structure-property patterns in diverse chemical spaces.^1^ Graph neural networks (GNNs) enable representation learning by directly operating on molecular graphs. In graph-based tasks such as property-based compound classification, graph pooling is an essential step for generating whole-graph representations from learned node embeddings.^2^ Graph pooling algorithms can generally be divided into two categories including global pooling and hierarchical pooling^2^. In global pooling, node embeddings are directly aggregated to yield a final graph representation. In hierarchical pooling^2^, the graph size is systematically reduced to generate intermediate representations that gradually capture the hierarchical structure of the input graph.^2^.

Several GNN architectures incorporating hierarchical pooling have been introduced.^3–5^ However, graph pooling generally depends on organizing and combining node information through complex graph-based operations. This makes it difficult to determine how many individual node features or subgraphs substantially contribute to the final graph representation and the internal operations and learning characteristics of the GNN model remain a black box. For explaining hierarchical pooling approaches, generally applicable evaluation metrics are currently not available.^6^ In drug discovery and design, lack of explainability and interpretability of model decisions generally restricts their acceptance, which also applies to GNNs and graph pooling. A previous study indicated superior performance of hierarchical pooling GNNs based on pharmacophore features in molecular property prediction compared to other methods.^4^ However, the learning characteristics of these GNNs remain underexplored.

We investigate hierarchical graph pooling by GNNs in molecular property prediction by focusing on explanations of model decisions. Therefore, we generate and compare three different GNN architectures including a standard graph attention (GAT) network ^7^, another GAT network trained on pharmacophore-based reduced graphs instead of complete molecular representations (GAT-rg), and a new pharmacophore-pooling graph attention network (PP-GAT) introduced in this work. PP-GAT is a hierarchical pooling network that reduces an input molecular graph to a pharmacophore-based graph via ad-hoc hidden pooling layers. Evaluation of model performance mostly relies on standard measures such as balanced accuracy or the area under the receiver operating characteristic curve (AUROC)^8^, which quantify prediction accuracy but cannot further differentiate between models. Therefore, we also employ various approaches from explainable artificial intelligence (XAI)^9,10^ to identify graph components driving GNN predictions and explore the learning behavior. Our analysis shows that in the presence of comparable predictive performance, learning characteristics and robustness of PP-GAT substantially differs from other GAT variants.

## Methods

### Compound data

For model evaluation, 10 target-based compound activity classes were selected from ChEMBL^11^(version 33), consisting of 3858 to 1305 active compounds. In each case, active compounds were complemented with an equal number of randomly selected ChEMBL compounds serving as inactive instances, resulting in balanced data sets. In addition to these activity classes, the models were also evaluated on other publicly available data sets including compounds with or without blood–brain barrier penetration (BBP), clinical toxicity (ClinTox), and anti-HIV activity (HIV), obtained from MoleculeNet^11^. BBP contained 1567 penetrating and 483 non-penetrating compounds, ClinTox 112 toxic and 132 non-toxic molecules, and HIV 1443 active and 39,684 non-active compounds.

### Graph construction

For the molecular graph representation, atom (node) and bond (edge) features were encoded as categorical (binary) or numeric physicochemical properties (Table 1). For graph reduction, the framework introduced by Kong et al.^4^ was implemented to condense the molecular graph into a pharmacophore-based reduced graph. The reduction process begins with assigning pharmacophore features including hydrogen-bond donors, acceptors, aromatic rings, aliphatic moieties, or ionizable groups. Following Kong et al., we also adopted the graph reduction scheme of Harper et al.^13^, which defines 18 types of pharmacophore nodes based on three categories of rings and six feature categories (Table 1). For generating the reduced graph, each atom was assigned to a pharmacophore group. Then, atom features were pooled within each group using mean value aggregation. Edges between pharmacophore nodes were derived from inter-group bonds, with bond multiplicity encoded as an edge attribute.

**Table 1:** Atom and bond features and pharmacophore node definition definitions.

Atom features	Atomic number, hybridization state, formal charge, valence, hydrogen atom number, aromaticity	
Bond features	Bond type, conjugation state, ring membership	
Ring category	Node feature category	Supernode symbol
Aromatic	Nonfeature	Sc
	Donor	Ti
	Acceptor	V
	Donor and acceptor	Cr
	Negatively ionizable	Fe
	Positively ionizable	Mn
Aliphatic	Nonfeature	Sc
	Donor	Ti
	Acceptor	V
	Donor and acceptor	Cr
	Negatively ionizable	Fe
	Positively ionizable	Mn
Acyclic	Nonfeature	Sc
	Donor	Ti
	Acceptor	V
	Donor and acceptor	Cr
	Negatively ionizable	Fe
	Positively ionizable	Mn

Listed are atom and bond features for graph representations and 18 types of pharmacophore node features for reduced graphs together with their so-called supernode symbols^13^. As supernode node symbols, pseudo-atom types were used^13^, making it possible to process and visualize reduced graphs with standard molecular modeling software.

### Model architectures

GAT is a GNN variant with attention mechanism^7^, enabling each node to assign different levels of importance to its neighbors. The network implemented in this work consists of four GATv2^14^ convolutional layers, with 64 hidden units implemented using PyTorch Geometric^15^. Each GAT layer is followed by an exponential linear unit (ELU) activation function. The final node embeddings are then aggregated via global mean pooling to obtain the molecule-level representation. The pooled vector is passed through two fully connected layers with intermittent rectified linear unit (ReLU) activation function to generate the final prediction logits. Output probabilities of the model are obtained by applying a sigmoid transformation to the final logits.

The GAT-rg model corresponds to the GAT model to enable direct comparison, with the exception of a different input representation, that is, reduced-pharmacophore graphs.

The newly introduced PP-GAT model extends the GAT architecture by modeling two hierarchical levels including the atom and pharmacophore level. This architecture was inspired by the message passing neural network (MPNN) model of Kong et al.^4^. Moreover, PP-GAT combines the attention mechanism with chemistry-aware graph representations, resulting in a novel architecture with the ability to learn and focus on specific chemical features for its predictions. It consists of two atom-level message passing layers, a graph reduction phase, in which the atoms are pooled into supernodes according to their pharmacophore group label (Table 1), and two pharmacophore-level message passing layers on the pharmacophore-reduced graph. The final node embeddings are aggregated via global mean pooling to obtain the molecule-level representation. In the reduced graph, bond multiplicity serves as an edge attribute analogously to the reduced graph in GAT-rg. PP-GAT enables explainability across hierarchical levels by dynamically constructing and reducing the graph within the network where pharmacophore-level edges are derived from the original atom-level connectivity. This links the reduced graph to the original structure during the pooling process, ensuring consistent explainability.

**Fig. 1:**
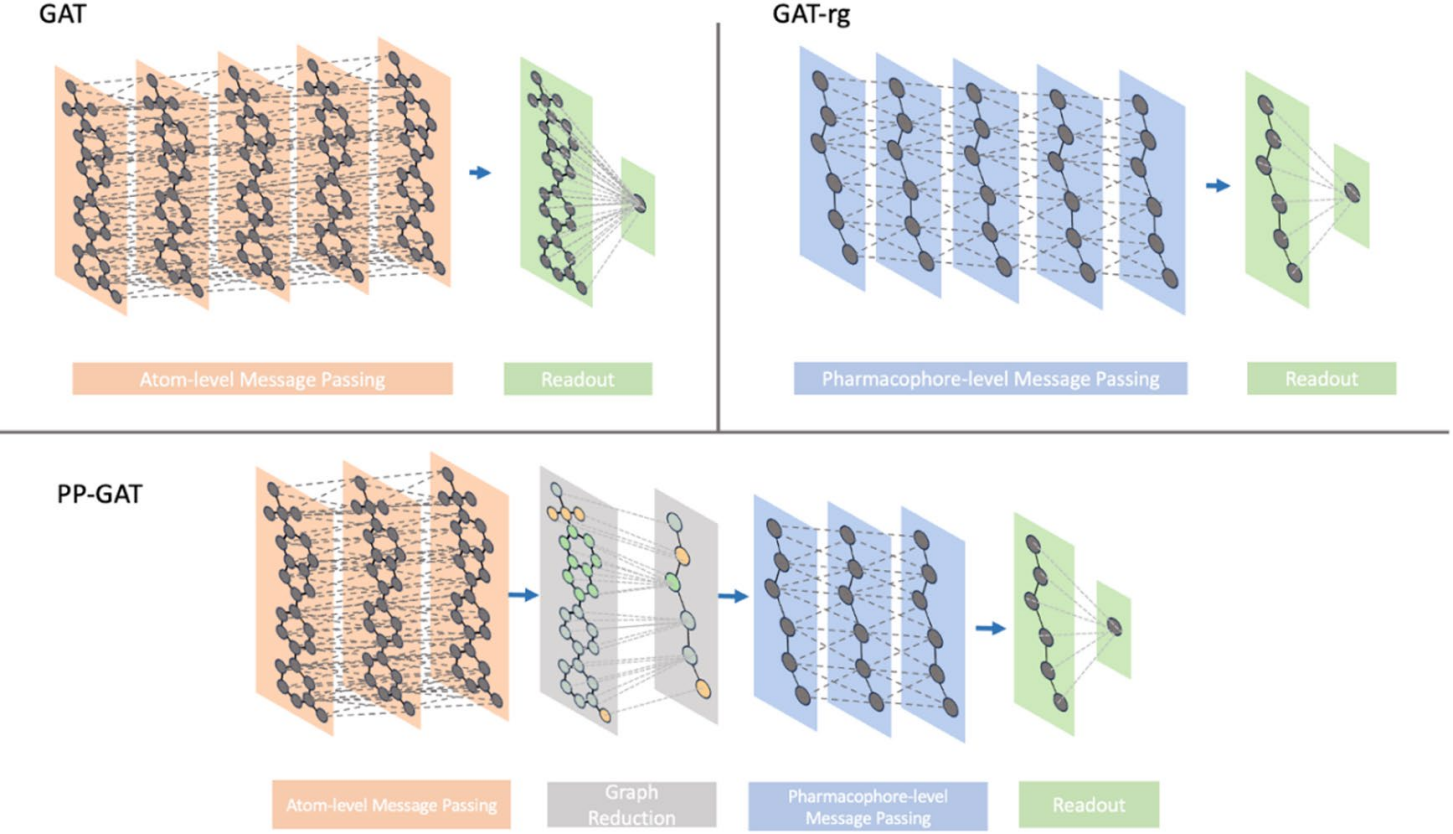
Graph neural network model architectures. The different model architectures of the GAT models analyzed in this study are schematically illustrated, including the reduced-graph models and the pharmacophore-pooling network.

Figure 1 illustrates the model architectures.

### Model derivation, optimization, and evaluation

Compound data sets were divided into training, validation, and test sets using a stratified 70/10/20% split ratio for hyperparameter optimization. For a constant data partition, hyperparameters were optimized via grid search over learning rates [1e−4, 5e−4, 1e−3] and batch sizes [16, 32, 64]. For each combination, a model was trained for 30 epochs using the Adam optimizer^16^ and a binary cross-entropy loss function with logits. The model with the lowest validation loss was selected. The final models corresponding to the best hyperparameter configurations were evaluated based on five independent trials, with balanced accuracy (BA) and AUROC as performance measures. For each trial, the data sets were randomly shuffled using a different random seed. Following shuffling, the sets were partitioned into 70% training data, 10% validation , and 20% test data. A single partition was generated for each independent trial and used for the GAT, GAT-rg, and PP-GAT models to ensure that all models were trained and evaluated on the same samples and that performance differences were only model-dependent.

### Model explanation

To investigate the learning characteristics of the models and rationalize their predictions, two distinct XAI approaches for GNNs were applied including GNNExplainer^17^ and EdgeSHAPer^18^. GNNExplainer aims to identify a minimal subgraph responsible for a GNN prediction. Subgraph identification is formulated as an optimization task to maximize the mutual information between the prediction based on the complete graph and a subgraph^17^. EdgeSHAPer quantifies the contribution of each edge in a molecular graph by estimating Shapley values^19^ using a Monte Carlo sampling strategy^18^. The key difference between GNNExplainer and EdgeSHAPer is that EdgeSHAPer can quantify positive or negative contributions of each edge in a graph to a GNN prediction.

Importantly, both GNNExplainer and EdgeSHAPer are applicable to reduced graphs and hierarchical model architectures (and hence to all GNN variants investigated herein), whereas other approaches such as Integrated Gradients^20^ or PGExplainer^21^ depend on a one-to-one semantic correspondence between input and output dimensions, which is not the case for PP-GAT. Similarly, chemistry-oriented fragment-based explanation methods such as MolAnchor^22^ require a complete molecular graph representation as input, different from reduced-graph approaches and hierarchical pooling that decomposes the original graph. In addition, the use of attention maps for model explanation, which is controversially viewed^23,24^, is not applicable to convey molecular graph information.

### Minimal informative sets

Building on the foundations of contrastive explanations (CE)^25,26^, we use the minimal informative set (MIS) formalism^26,27^ to identify the smallest structural subsets guiding a model’s prediction. By integrating the logic of pertinent positive sets^26^ with Shapley-based edge ranking^18^, three sets were defined including the pertinent positive set (P_Pos_)^26,27^, the minimal top-k set (TK)^18^, and the minimal opposing set (MO). P_Pos_ represents the minimal number of edges sufficient to yield the original prediction and TK the minimal number of top-ranked edges to be removed from the graph to invert the predicted class. Hence, P_Pos_ describes the minimal substructure required to generate the prediction, highlighting the structural elements the model relies on. On the other hand, TK provides insights into the robustness of a prediction. Furthermore, as introduced herein, MO provides the smallest number of supporting edges that need to be added to all opposing edges to recover the original prediction. MO set identification process begins with a graph containing only edges that oppose a given prediction. Then, edges that support the prediction are added incrementally according to the magnitude of their positive contributions until the original prediction is recovered. Accordingly, MO accounts for the amount of positive evidence required to compensate for the present negative evidence and thus provides insights into the balance of positive and negative contributions to a prediction.

## Results and discussion

### Model performance

The prediction accuracy of GAT, GAT-rg, and PP-GAT models for compound classification using 13 different data sets was systematically determined. Table 2 reports the results over five independent trials for the 10 activity classes from ChEMBL (balanced composition of active and inactive compounds) and the BBP, ClinTox, and HIV data sets from MoleculeNet (unbalanced) .

**Table 2:** Prediction accuracy for activity classes and datasets.

Activity class	Model	BA	AUROC	Positive/negative samples
Hepatocyte growth factor receptor (P08581)	GAT GAT-rg PP-GAT	0.94 ± 0.01 0.93 ± 0.01 0.91 ± 0.02	0.98 ± 0.00 0.97 ± 0.00 0.96 ± 0.01	1414
Vascular endothelial growth factor receptor 2 (P35968)	GAT GAT-rg PP-GAT	0.87 ± 0.01 0.87 ± 0.01 0.86 ± 0.02	0.94 ± 0.01 0.94 ± 0.00 0.92 ± 0.01	2523
Carbonic anhydrase 9 (Q16790)	GAT GAT-rg PP-GAT	0.97 ± 0.01 0.95 ± 0.01 0.96 ± 0.01	0.99 ± 0.00 0.98 ± 0.00 0.99 ± 0.00	3779
Beta-secretase 1 (P56817)	GAT GAT-rg PP-GAT	0.92 ± 0.02 0.91 ± 0.02 0.94 ± 0.01	0.97 ± 0.01 0.96 ± 0.01 0.98 ± 0.01	1380
Acetylcholinesterase (P22303)	GAT GAT-rg PP-GAT	0.91 ± 0.01 0.90 ± 0.01 0.90 ± 0.01	0.96 ± 0.01 0.95 ± 0.01 0.95 ± 0.01	2068
Cholinesterase (P06276)	GAT GAT-rg PP-GAT	0.90 ± 0.01 0.90 ± 0.01 0.91 ± 0.01	0.96 ± 0.01 0.96 ± 0.01 0.96 ± 0.01	1305
Carbonic anhydrase 1 (P00915)	GAT GAT-rg PP-GAT	0.97 ± 0.00 0.96 ± 0.01 0.96 ± 0.00	0.99 ± 0.00 0.98 ± 0.01 0.99 ± 0.00	3858
Bifunctional epoxide hydrolase 2 (P34913)	GAT GAT-rg PP-GAT	0.95 ± 0.01 0.94 ± 0.01 0.94 ± 0.01	0.99 ± 0.00 0.98 ± 0.00 0.98 ± 0.00	1442
Histone deacetylase 1 (Q13547)	GAT GAT-rg PP-GAT	0.97 ± 0.01 0.96 ± 0.00 0.96 ± 0.00	0.99 ± 0.00 0.98 ± 0.00 0.98 ± 0.00	2028
Dipeptidyl peptidase 4 (P27487)	GAT GAT-rg PP-GAT	0.97 ± 0.01 0.96 ± 0.01 0.96 ± 0.01	0.99 ± 0.00 0.98 ± 0.00 0.98 ± 0.00	1740
BBP	GAT GAT-rg PP-GAT	0. 78 ± 0.03 0.80 ± 0.02 0.79 ± 0.02	0.87 ± 0.02 0.87 ± 0.01 0.86 ± 0.02	1567/483
ClinTox	GAT GAT-rg PP-GAT	0. 69 ± 0.05 0.58 ± 0.04 0.67 ± 0.04	0.89 ± 0.03 0.77 ± 0.03 0.86 ± 0.03	112/132
HIV	GAT GAT-rg PP-GAT	0. 62 ± 0.02 0.64 ± 0.02 0.64 ± 0.01	0.77 ± 0.02 0.77 ± 0.02 0.77 ± 0.02	1443/39,684

For each activity class, the target name and ChEMBL target identifier (in parentheses) are provided. Reported are the mean and standard deviation. Positive samples indicate the number of active compounds (activity classes and HIV data set), penetrating molecules (BBP), and toxic compounds (ClinTox). For the three unbalanced data sets, the number of negative (inactive, non-penetrating, or non-toxic) compounds is also reported.

The performances of the three models across the five independent runs were compared using the Wilcoxon signed-rank test^28^. For both AUROC and BA, no statistically significant differences were observed between any pair of models across all (all *p*-values were greater than 0.05).

For the different activity classes, prediction accuracy was consistently very high for all three GNN variants, with AUROC values > 0.9 in all cases and BA values > 0.9 for nine of 10 classes (and one with BA ~ 0.86–0.88). For BBP, prediction accuracy was only slightly reduced with AUROC values > 0.8 and BA values  ~ 0.79. For HIV, AUROC and BA values were slightly further reduced (~ 0.77 and ~ 0.62–0.64, respectively). For ClinTox, all models had lower BA (~ 0.58–0.68). In addition, standard deviations over independent trials were consistently very low, indicating stable predictions, and there were only minimal variations in performance for the GNN model variants. However, the consistently high performance of PP-GAT was a notable finding, indicating that pharmacophore-based graph reduction retained essential property-related information present in complete molecular graphs.

**Fig. 2:**
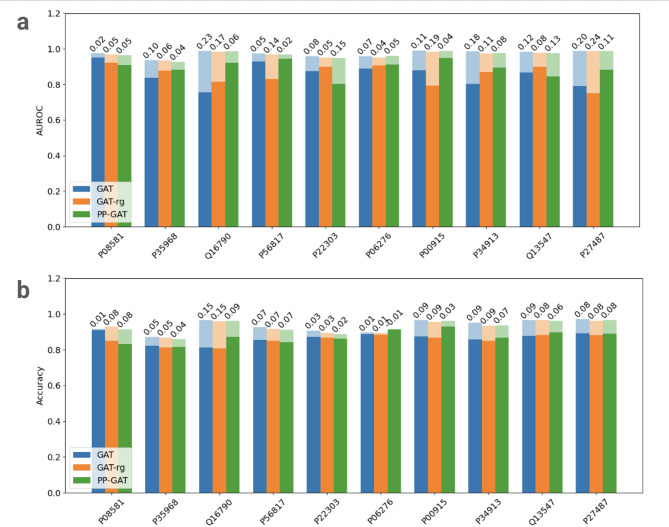
Model performance for random vs. analogue series-based data partitioning. For all activity classes and models, (**a**) and (**b**) show AUROC and BA values, respectively, as the mean over five independent trials. Corresponding darker and lighter colors represent model performance for analogue series-based and random data splits, respectively. The difference between the mean values is reported above the bar for random splits. The positive differences indicate higher model performance for random splits.

In addition to models based on random data splitting, we tested the GNN variants following analogues series-based data partitioning, which corresponds to scaffold-based splitting^29^. An analogue series comprises compounds that share the same core but have different substituents. In random compound partitioning, closely related compounds (analogues) are likely to occur in training and test data, supporting learning and predictions. In analogue series-based splitting, complete analogue series are assigned to either training or test sets, thus preventing the presence of highly similar compounds in training and test data. Accordingly, analogue series-based partitioning controls for potential structural data leakage. Therefore, we systematically extracted analogue series from all 10 activity classes and retrained the GNN variants following analogue series-based partitioning of training and test data. Figure 2 compares the results for the models based on random and analogue series-based partitioning.

As expected, prediction accuracy was generally higher for random compared to analogue series-based data partitioning , albeit only by small margins. Both pharmacophore-based models achieved slightly higher classification accuracy than GAT, and for seven of the 10 activity classes, PP-GAT performed best. However, while consistent, none of the observed differences was statistically significant, as revealed by the Wilcoxon signed-rank test (no *p*-value was smaller than 0.05). Thus, predictive performance was overall not significantly affected by potential data leakage.

We then analyzed individual predictions in more detail, searching for test compounds with different prediction outcomes (correct/incorrect) of PP-GAT compared to GAT/GAT-rg. For instance, for activity class Q16790 (carbonic anhydrase 9 inhibitors), we identified 15 active compounds that were correctly predicted by PP-GAT but misclassified by GAT and 11 other compounds misclassified by PP-GAT but correctly predicted by GAT. In the presence of closely comparable prediction accuracy, such inconsistent predictions can be used to further explore learning characteristics of different models, here especially the influence of pharmacophore-based hierarchical pooling on model decisions.

### Explaining predictions based on feature attributions

**Fig. 3:**
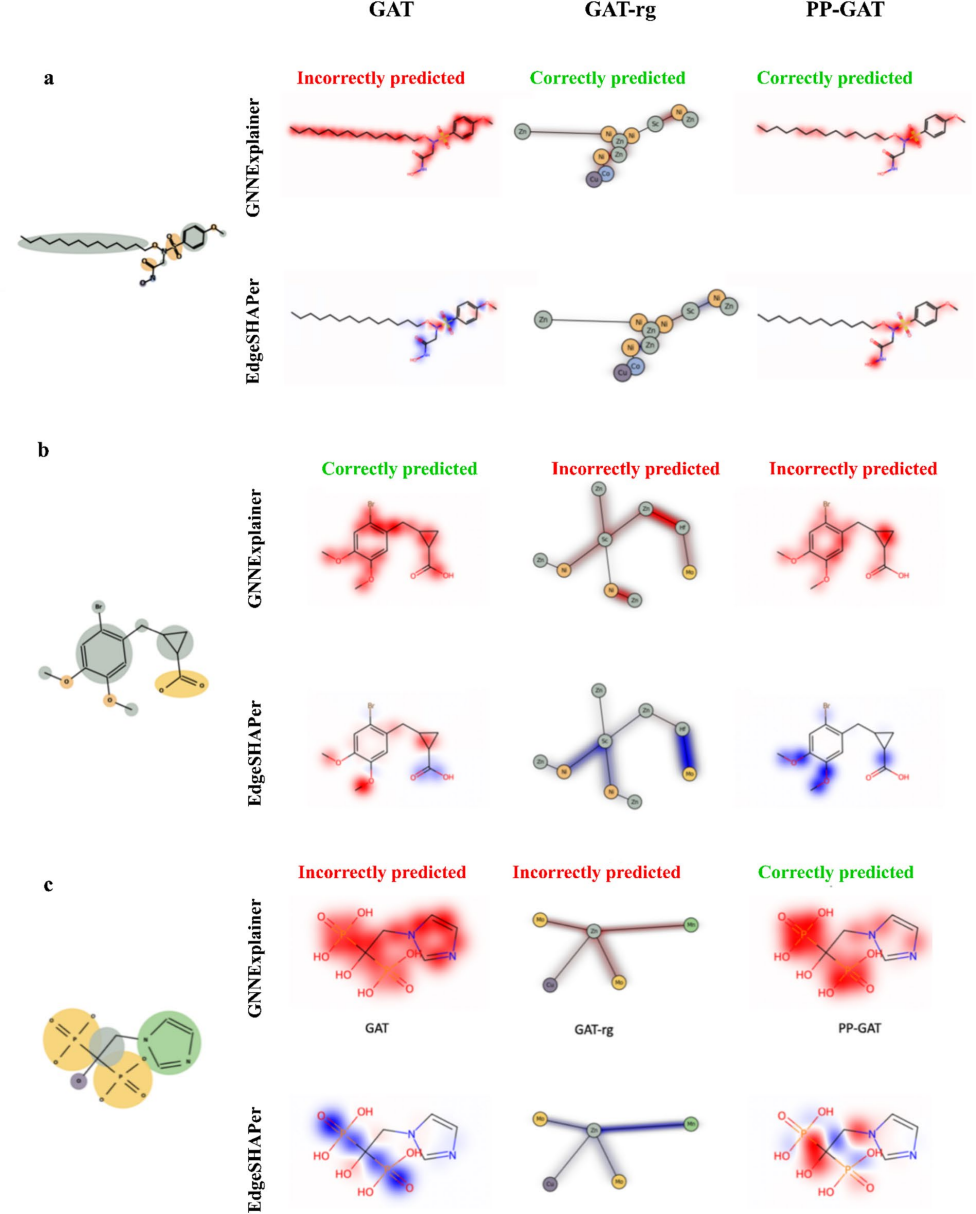
Feature mapping. In (**a**–**c**), prediction results and feature mappings are shown for three exemplary compounds. For GNNExplainer, red shading indicates positive contributions to the predicted class. For EdgeSHAPer, red shading indicates positive (supporting) contributions to the correct prediction of activity, whereas blue shading indicates negative (opposing) contributions. Color intensity reflects the magnitude of the contributions. For the reduced graph visualizations, nodes represent pooled substructures, rather than individual atoms. On the left, correspondence between reduced-graph nodes and the original molecular graph is illustrated where regions shaded using different colors define the atoms grouped into each reduced node.

Given the very consistent prediction accuracy of the different models on all data sets, we selected two exemplary activity classes for in-depth XAI analysis, which required the evaluation of many individual predictions. These classes included inhibitors of carbonic anhydrase 9 and histone deacetylase 1 and were selected based on the number of incorrectly predicted compounds, as stated above, and due to the wealth of structure-activity relationship information that is available for these inhibitors . Using GNNExplainer and EdgeSHAPer, feature importance for individual predictions was quantified and features contributing to the predictions were mapped on compound structures for visualization. Figure 3a shows the results of feature mapping for an exemplary carbonic anhydrase 9 inhibitor incorrectly classified as inactive by GAT, but correctly classified as active by both GAT-rg and PP-GAT. Hence, in this case, graph pooling led to a correct prediction. For the standard GAT model, GNNExplainer highlighted nearly the entire compound structure including the long aliphatic chain to contribute to the incorrect prediction (red shading), whereas EdgeSHAPer focused on contributions of individual atom or groups leading to the incorrect prediction (blue shading), excluding the long aliphatic chain. Thus, these alternative explanations were inconsistent. By contrast, for the correct predictions by both the GAT-rg and PP-GAT model, GNNExplainer and EdgeSHAPer revealed largely corresponding contributions in the core of the compound, excluding the aliphatic chain, with the exception of the sulfone group that supported the correct prediction based on GNNExplainer attributions, but opposed it based on EdgeSHAPer attributions. However, the correspondence of various feature contributions suggested that graph pooling propagated activity-relevant information for individual atoms or groups. Furthermore, these patterns indicated that graph pooling shifted feature importance to higher-level structural features obtained by pharmacophore-based graph reduction.

Figure 3b shows another carbonic anhydrase 9 inhibitor that was correctly classified by GAT, but incorrectly by GAT-rg and PP-GAT. Here, GNNExplainer attributions also assigned importance to essentially the entire molecular structure for the correct prediction. However, pharmacophore-based graph reduction centered most of the importance on two substructures. By contrast, EdgeSHAPer emphasized individual feature contributions including strong positive contributions of one of the two methoxy groups and a cyclopropane ring in the compound, thus providing a more differentiated picture. However, EdgeSHAPer explanations of the incorrect prediction of the PP-GAT model indicated strong contributions of both methoxy groups to the prediction and essentially no contribution of the cyclopropane ring. Thus, in this case, abstraction from the original molecular structure through pharmacophore pooling, merging the cyclopropane ring into a single node, might cause a loss in structural information and the corresponding contribution to the prediction.

In Fig. 3c, an inhibitor is shown that was only corrected predicted by the PP-GAT model based on pharmacophore and hierarchical graph pooling (hybrid pooling). Based on EdgeSHAPer explanations, the phosphate groups in this compound strongly contributed to incorrect predictions by the GAT and GAT-rg models. By contrast, GNNExplainer and EdgeSHAPer explanations indicated that both phosphate groups or one of these groups, respectively, made strong contributions to the correct prediction of the PP-GAT model. Accordingly, this comparison revealed a model-dependent inversion of specific contributions to predictions. These contributions can be rationalized based on known structure-activity relationships. Phosphate groups in the given inhibitors complex the zinc cation in the active site of carbonic anhydrase, which is essential for catalytic activity. Thus, in this case, the explanations for the PP-GAT model correctly identified a key feature for enzyme inhibition that determined the correct PP-GAT prediction and clearly distinguished between the GAT/GAT-rg models and PP-GAT. Notably, hybrid pooling integrates information from the original molecular graph and the pharmacophore-based reduced graph. While graph reduction might lead to a loss of detailed structural information, hierarchical pooling might compensate for this loss by focusing on the most important graph components for the predictions. Depending on the test compounds and their activity determinants, such as specific groups and/or molecular shape, hybrid pooling might support or impair predictions of individual compounds, as indicated by the compound examples discussed above. We recall that global prediction accuracy of the GNN variants was comparably high and that inconsistent predictions were only detected for small subsets of the test compounds. However, explanations of individual inconsistent predictions can uncover different learning behavior of the model variants, as illustrated by the compound examples.

**Fig. 4:**
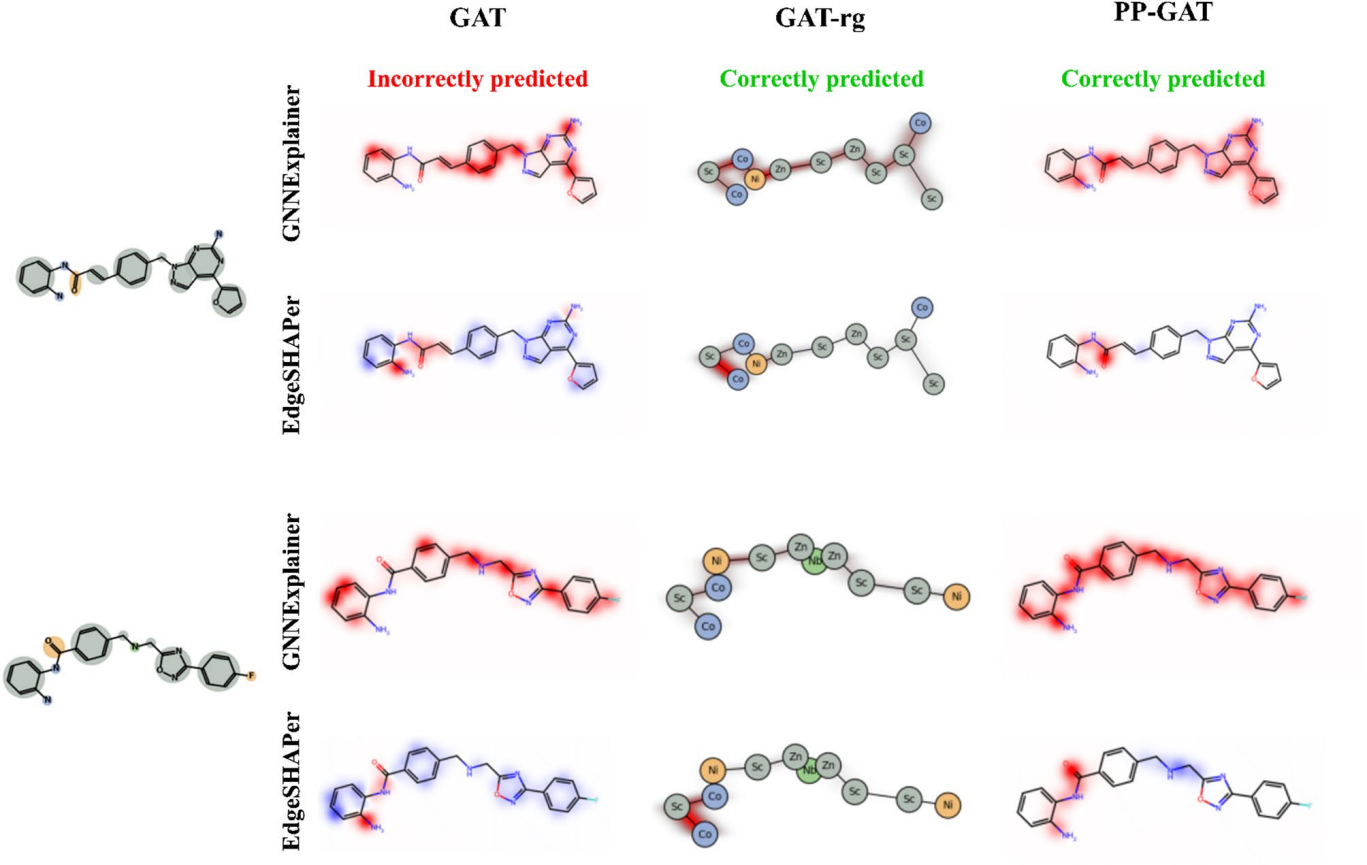
Functional group-based explanations in reduced and pooled models. Shown are four exemplary inhibitors that were correctly predicted by PP-GAT and GAT-rg, but misclassified by the standard GAT model. The presentation is according to Fig. 3.

In another activity class, inhibitors of histone deacetylase 1 (Q13547), 60 active compounds were misclassified by GAT, but only one was incorrectly classified by the PP-GAT model. Thus, to further analyze effects of pharmacophore-based hierarchical pooling, we also compared predictions for exemplary test compounds belonging to this class. Figure 4 shows explanations for histone deacetylase 1 inhibitors.

A strong positive contribution of the carbonyl group (of the original hydroxamic acid moiety) to the prediction of activity was consistently detected for the PP-GAT model in these inhibitor derivatives, but not for the GAT model when incorrectly predicting these compounds. This functional group is contained in many inhibitors and binds to the center of the active site in histone deacetylase, consistent with its positive contribution to the correct predictions of the PP-GAT model. In contrast to GAT, accurate predictions of these inhibitors by the PP-GAT and GAT-rg models also indicated that the reduced and pooled representations supported the detection of this conserved small functional group as a hallmark of activity, which was likely due to noise reduction in less relevant parts of the compounds and the focus on relevant important pharmacophore features. In less relevant regions, GAT distributed varying contributions across a larger number of nodes and edges, which frequently led to incorrect predictions.

### Explaining predictions using minimal informative sets

To further extend the qualitative analysis of inconsistent predictions in more quantitative terms, we carried out MIS analysis to better understand how the different GNN variants arrived at their decisions. Therefore, we reinvestigated carbonic anhydrase 9 inhibitors because for this activity class, a comparable number of compounds was misclassified by GAT and PP-GAT. Specifically, we determined the average number of edges in P_Pos_, TK, and MO sets for 100 active and 100 inactive randomly selected compounds that were consistently correctly predicted by all three models. Table 3 reports the results.

**Table 3:** Mean minimal informative sets.

Model	*P*_Pos_	TK	MO	Supporting edges	Opposing edges	Expected value
Active						
GAT	2.83	2.55	8.33	23.58	32.16	0.45
GAT-rg	1.00	3.00	2.57	9.15	7.87	0.70
PP-GAT	1.02	2.16	2.48	24.34	31.40	0.80
Inactive						
GAT	1.00	9.49	9.66	17.18	49.80	0.44
GAT-rg	5.70	4.26	5.20	5.10	14.08	0.64
PP-GAT	1.68	6.82	4.53	28.68	38.30	0.68

For the three models, the average number of edges in the P_Pos_, TK, and MO sets is reported for 100 active and 100 inactive consistently correctly predicted test compounds. The expected value was calculated as the average prediction on the random graphs forming the information background sample for Shapley value calculations using EdgeSHAPer.^18^.

For active compounds, both the GAT-rg and PP-GAT models had P_Pos_ values of ~ 1, indicating that a single strongly supporting edge was sufficient for the original prediction; a surprising finding. However, the high expected values of these models suggested that the expected values were already sufficient for correct inference, indicating a potential bias towards active compounds. However, GAT required a slightly larger P_Pos_, despite having fewer supporting edges overall, indicating contributions from multiple features. A different pattern was observed for inactive molecules. Here, P_Pos_ was larger for GAT-rg, with ~ 5.7 edges, showing that prediction of inactivity required more edges, which was a likely consequence of the larger diversity of inactive compared to active compounds, particularly mirrored by reduced graphs.

Considering TK sets, for active molecules, we observed an apparent balance (~ 2.2–3.0 edges) between the models. However, the total number of edges was smaller for GAT-rg. Accordingly, GAT-rg required the removal of a larger ratio of supporting edges to invert the prediction, indicating that it was less sensitive to edge removal and that its predictions were more robust.

For both active and inactive molecules, MO sets for GAT were larger than for PP-GAT, showing that the addition of a substantial number of edges with positive contributions was required to compensate for the presence of opposing edges and recover the original prediction, different from PP-GAT. Notably, for inactive molecules, the MO set of GAT-rg was comparable in size to the total number of supporting edges, hence indicating that the total positive evidence was required to compensate for the edges making negative contributions. Taken together, these results reflected internal differences and trade-offs in learning characteristics of the GAT model variants.

### Feature ablation analysis

To explore potential effects of the different learning characteristics, we then carried out an ablation analysis for the input molecular graphs to identify components that were indispensable for effective learning. Therefore, we compared the performance of the three models after removal of node features (while retaining edges) and after removal of edges (retaining node features with no connections). The configuration after removal of edges served as an effective baseline control for evaluating the discriminative hybrid pooling approach. This was the case because the absence of edges prevented message passing, for which edges are crucial, both within the original graph and the internal pooled representation. Table 4 reports the results.

**Table 4:** Ablation study.

Model	Complete graph		No node features		No edges	
	AUROC	BA	AUROC	BA	AUROC	BA
GAT	0.99 ± 0.00	0.97 ± 0.00	0.50 ± 0.00	0.50 ± 0.00	0.50 ± 0.00	0.50 ± 0.00
GAT-rg	0.99 ± 0.00	0.96 ± 0.00	0.50 ± 0.00	0.50 ± 0.00	0.50 ± 0.00	0.50 ± 0.00
PP-GAT	0.99 ± 0.01	0.96 ± 0.01	0.50 ± 0.00	0.50 ± 0.00	0.97 ± 0.00	0.92 ± 0.01

The performance of the original model is compared to the performance of models derived in the absence of node features or edges. Mean performance and standard deviation over five independent trials are reported.

After removal of node features, all models failed, yielding random prediction accuracy. In the absence of edges, GAT and GAT-rg models also had random accuracy. By contrast, PP-GAT retained high predictive performance (with AUCROC and BA values of 0.97 and 0.92, respectively). Thus, while node features were essential for all predictions, the hybrid pooling mechanisms of PP-GAT, which distinguished it from GAT and GAT-rg, was able to compensate for the absence of edge information. A likely reason for this ability was the implicit encoding of connectivity information via the pooling procedure. This encoding scheme embedded connectivity information in the pharmacophore features, which can be rationalized as an implicit message passing step, thus compensating for edge-dependent message passing. However, this required the pooling groups to reflect the original graph structure.

## Conclusions

In this work, we have analyzed the predictive performance and learning characteristics of pharmacophore-based GAT models compared to the original GAT architecture. While previous work^4^ suggested benefits of incorporating pharmacophore representations in GNN-based molecular property prediction, our results did not reveal a significant advantage of pharmacophore-based graph reduction and/or hierarchical pooling in compound activity predictions compared to GAT. However, the ability of reduced graph-based GAT models to match the prediction accuracy of models based on complete graph representation was a notable finding, indicating that pharmacophore-based reduced graphs retained key structural features required for accurate predictions. With PP-GAT, we also introduced a new pharmacophore-pooling graph attention network that reached the same performance level as the other GNN variants in activity predictions with random or analogue series-based data partitioning. Hybrid pooling, as introduced herein, represents the characteristic component of PP-GAT. In the presence of comparable prediction accuracy, we then explored learning characteristics of the GNN variants. Initially, qualitive feature attribution and mapping analysis was carried out for inconsistent predictions, followed by quantitative feature analysis for consistently accurate predictions based on the MIS formalism. Explanations of individual predictions revealed different -and often opposing- feature contributions that were prioritized by the GNN variants for their predictions. Furthermore, MIS analysis conclusively showed that model decisions were primarily driven by small feature sets of varying composition. These findings revealed different learning characteristics of the GNN model variants that were further explored by a feature ablation analysis. Elimination of node features led to random prediction accuracy of all models. Thus, node features were critically important for the predictions. Elimination of edge information equally reduced prediction accuracy of the GAT and GAT-rg models. By contrast, despite edge elimination, PP-GAT retained high prediction accuracy as a consequence of its hybrid pooling mechanism. These findings further extended the identification of different learning behavior of the GAT variants.

## Data Availability

Data and code generated and used in this study are available on GitHub at https://github.com/LSI-Uni-Bonn/Pharmacophore-Pooling-Graph-Attention-Network and on Zenodo at 10.5281/zenodo.19738381.
